# Dynamic Assessment as a Self-Regulation Strategy in the Acquisition of Textual Revision

**DOI:** 10.3390/bs16010123

**Published:** 2026-01-15

**Authors:** Olga Arias-Gundin, Celestino Rodríguez, Raquel Fidalgo

**Affiliations:** 1 Psychology, Sociology and Philosophy Department, University of León, 24071 León, Spain; rfidr@unileon.es; 2 Department of Psychology, University of Oviedo, 33003 Oviedo, Spain; rodriguezcelestino@uniovi.es

**Keywords:** dynamic assessment, self-regulation, textual revision, writing, secondary education

## Abstract

Textual revision is a recursive process integral to writing. However, less experienced writers often struggle to select effective strategies, underuse self-regulation, and evaluate their work without metacognitive control. This study examined the effectiveness of instructional programs focused on textual revision, incorporating dynamic assessment as a means to promote self-regulation. A total of 88 secondary school students (aged 13–15) participated, randomly assigned by class group to one of four conditions: mechanical revision, substantive revision, combined revision, or rewriting. A quasi-experimental design with repeated measures was used to assess the revisions carried out by the students Each intervention focused on distinct revision strategies: surface-level corrections, content and structure, or a combination of both. The rewriting group received no specific instruction beyond the weekly practice of rewriting the text that the other groups worked on. Findings revealed that students in the substantive revision group achieved the greatest gains in their revisions. The study concludes that instructional approaches focused on deep, content-oriented revision are particularly effective in improving students’ writing performance and fostering self-regulatory skills. These findings highlight the value of embedding metacognitive support in revision-focused instruction.

## 1. Introduction

Writing constitutes a fundamental pillar of human communication and a key skill for personal, academic, professional, and social development (Harris & McKeown, 2022). Beyond its instrumental function, writing represents a powerful tool for organizing thought, engaging in critical reflection, constructing knowledge, and participating in civic and cultural life (Hayes, 2012). In academic contexts, writing competence is closely associated with higher-order thinking, school success, and access to further educational opportunities. However, developing this competence requires much more than mastering grammar or syntax; it demands engagement in recursive, metacognitive, and self-regulatory processes that are often demanding and permeate all phases of text production (Torrance et al., 2022).

Drawing on major cognitive models of writing (Berninger & Amtmann, 2003; Bereiter & Scardamalia, 1987; Graham, 2018; Hayes, 2012; Hayes & Flower, 1980; Kellogg, 1996), research typically distinguishes between two broad categories of cognitive processes involved in writing: lower-level processes and higher-order processes. The former corresponds to transcription skills, which enable writers to translate ideas into written language. Although these processes tend to become automated with experience, they still impose a cognitive load even on adult writers (Bourdin & Fayol, 2002), and this load is particularly heavy at younger ages, when considerable cognitive resources are allocated to orthographic and motor control (Fayol, 1999). As students’ progress through schooling and gain fluency, transcription gradually becomes automatized, such that skilled writers require minimal conscious attention to carry it out (Kellogg, 2008). Higher-order cognitive processes, in turn, encompass planning and revising, both understood as essential self-regulatory skills for writing. Planning supports the generation, organization, and prioritization of ideas in accordance with textual demands and communicative purposes, whereas revision enables writers to evaluate their texts, detect problems, and implement local or global modifications. Together, these processes regulate the act of writing and have been shown to be strong predictors of writing performance (Limpo & Alves, 2013, 2018). However, their relative influence appears to vary across educational levels: planning emerges as a particularly relevant predictor in the early years of compulsory schooling, whereas revision demonstrates a clearer contribution to text quality in later grades (Cordeiro et al., 2019; Limpo et al., 2014). As with transcription, research has demonstrated that text quality can be improved through explicit instruction in both planning (Arrimada et al., 2019; MacArthur et al., 2015) and revision (MacArthur, 2016) at different educational stages.

Planning and revision are supported by two overarching families of higher-order skills: metacognitive skills, entailing the monitoring, evaluation, and regulation of one’s cognitive processes during writing (e.g., monitoring progress towards goals, identifying mismatches between intention and written output), and self-regulatory skills, which involve managing motivation, effort, strategic behavior, and persistence (Zimmerman & Schunk, 2011). Although closely related, metacognition and self-regulation refer to different processes: metacognition concerns knowledge and monitoring of cognition, whereas self-regulation involves active control of behaviors, emotions, and cognition in relation to writing goals. Empirical research has shown that both are key predictors of text quality (Zimmerman & Kitsantas, 1999), and that writers who explicitly monitor and regulate their strategies tend to produce more coherent and better-structured texts.

As noted earlier, among the phases or processes involved in writing, text revision plays a particularly important role. Far from being a final or superficial step, revision is a critical dimension of the writing process that enables writers to refine ideas, adjust the organization of content, improve coherence, and ensure that the text is well aligned with its purpose and audience (Arias-Gundín & García-Sánchez, 2006; Graham, 2018; Hacker, 2018). Revising is not merely correcting; it is transforming. Effective revision requires identifying problems, generating alternatives, and making strategic decisions to improve the text, simultaneously mobilizing cognitive, motivational, and metacognitive resources (McCutchen, 2011). However, revision is intrinsically complex. It operates at multiple levels, from surface-level corrections such as spelling or grammar (mechanical revision) to deeper changes affecting content, structure, or meaning (substantive revision) (Alamargot & Chanquoy, 2001). It also demands mastery of a wide repertoire of strategies: deleting, reorganizing, expanding, reformulating, or even restructuring entire sections of text (Chanquoy, 2009). Given the limits of cognitive and attentional resources, some processes must be automatized to reduce cognitive load. Thus, whereas mechanical revisions tend to be automatized in expert writers, substantive revisions, being deeper and more controlled, interrupt the flow of other processes. These operations require not only declarative knowledge of writing conventions but also procedural knowledge about how to apply them in specific contexts and conditional knowledge about when these applications are appropriate. Writers must also monitor their progress, evaluate alternatives, and self-regulate their cognitive effort, an essential feature of self-regulated learning (Zimmerman & Schunk, 2011).

Despite its importance, research has consistently shown that novice or inexperienced writers rarely engage in revision spontaneously or effectively (Cuenat, 2022; López et al., 2019), highlighting the need for instructional intervention and scaffolding (Perez, 2001). When they do revise, they tend to focus on surface-level issues while overlooking deeper problems related to content or organization (Hacker et al., 1994; Piolat & Roussey, 1991). This limitation should not be interpreted as a lack of interest or ability, but rather as the result of inadequate strategy selection, limited procedural knowledge, and insufficient use of metacognitive and self-regulatory monitoring (Graham, 1997; Graham et al., 1993; Reder & Schunn, 1996; Wong, 1998, 1999). Consequently, beginning writers require explicit and scaffolded instruction to understand what revision entails, how it can improve their writing, and how to carry it out strategically. From this perspective, revision is not merely a writing skill but a self-regulated process involving planning, monitoring, evaluating, and adapting strategies in accordance with communicative goals (Zimmerman & Kitsantas, 1999, 2002). It should therefore be taught as a dynamic and context-sensitive process rather than a simple checklist. Instruction in revision must address both its cognitive and metacognitive dimensions and promote learners’ autonomy in decision-making about their own texts.

Within this framework, dynamic assessment emerges as a particularly relevant instructional approach, functioning as a multilevel support system that adapts scaffolding to the specific needs of each learner (Sink, 2016; Utley & Obiakor, 2015). This approach makes it possible to estimate learning potential by comparing learners’ independent performance with the level they reach when provided with graduated assistance, thus establishing the distance between these two levels as an indicator of potential development (Orrantía et al., 1997, 1998). From this perspective, dynamic assessment plays an essential role as a complementary tool to educational practice, facilitating the identification of necessary adjustments in teaching procedures, resources, and learning environments (Spector, 1992).

A key contribution of dynamic assessment is that it enables the evaluation not only of what students can achieve independently but also of what they can accomplish with scaffolded support (Lidz & Elliott, 2000). Assessment should therefore not be limited to identifying already consolidated knowledge and skills (i.e., the learner’s actual developmental level), but should seek primarily to analyze competencies still in development, aligned with the learner’s potential level. This form of assessment offers insight into learning processes, since completing a task with support does not indicate an absence of competence but represents an intermediate stage in skill acquisition, in which guided assistance is essential for progress (Marco & Cubero, 1997).

This approach aligns with the principle of individualized instruction, acknowledging that learners differ in prior knowledge, cognitive styles, and responsiveness to feedback. By integrating assessment and instruction, dynamic assessment enables the provision of tailored scaffolds that foster cognitive modifiability and the internalization of revision strategies.

Despite its theoretical and practical relevance, few studies have examined the effects of instructional programs on text revision that simultaneously integrate a focus on rewriting, self-regulation, and a multi-tiered system support such as dynamic assessment. The present study aims to address this gap by analyzing the impact of a specific instructional intervention on students’ revision practices and their ability to approach revision as self-regulated learners. In particular, programs evaluate the extent to which differentiated instruction increases the number of revisions performed by secondary school students, and in which aspects, mechanical, substantive, or both. Accordingly, this study is guided by the following hypotheses: (i) program focusing exclusively on mechanical aspects of revision is expected to lead to a greater number of revisions at this level (spelling, grammar, and punctuation), given that Spanish secondary school students have already acquired declarative knowledge in these areas, such as orthographic, grammatical, and punctuation rules; although they may still lack the procedural and conditional knowledge required to activate the revision process; (ii) programs addressing the substantive aspects of revision are expected to enable students both to activate the revision process and to focus their revisions on the text content, increasing the number of revisions made in order to reorganize, add, delete, or modify content; and finally, and (iii) the most effective program, which will increase the number of revisions at both mechanical and substantive levels, will be one that integrates instruction on both mechanical and substantive aspects of revision, helping students automatize the former while directly and explicitly addressing the latter.

## 2. Materials and Methods

### 2.1. Participants

A total of 88 students from a state secondary school in the province of León participated in the present study. The sample consisted of 51.14% female students (*n* = 45) and 48.86% male students (*n* = 43). All participants were enrolled in the second year of Compulsory Secondary Education, aged between 13 and 15 years, with the distribution presented in Table 1. None of the students had identified special educational needs, and their curricular competence levels fell within the parameters established by current national legislation for this educational stage (see National Royal Decree 217, 2022). Most students came from families with medium income levels, according to school records. Nevertheless, 79 students completed the study; that is, they participated both in the nine sessions that comprised the instructional program and in the two assessment sessions. In Table 1, the final sample is reported in parentheses.

For the implementation of the instructional programme, which was carried out during the second and third terms of the academic year, all four natural class groups of the school were involved. Each of these groups was randomly assigned to one of the instructional conditions defined in the study design.

### 2.2. Design

The study employed a quasi-experimental design with repeated pretest–posttest measures, involving four groups. Each group, within its regular classroom context and routines, received a different type of intervention: three groups were instructed in one of the designed modalities of the instructional and self-regulated revision program (mechanical, substantive, or combined), while the fourth group engaged in text rewriting practices. In all cases, the dependent variables were measured before and after the intervention, both through the Written Composition Revision Instrument (Instrumento de Revisión de la Composición Escrita, IRCE, by its Spanish acronym) (Arias-Gundín & García, 2007). These measures allowed for the assessment a detailed analysis of the number of revisions made by students, both at the mechanical level and at the substantive level. This design enabled the examination of the differential impact of each intervention modality on the number of revisions made by students in an authentic educational context.

### 2.3. Assessment

To evaluate the effectiveness of the intervention, we employed the rewriting task from the Written Composition Revision Instrument (IRCE, by its Spanish acronym of Instrumento de Revisión de la Composición Escrita), which has shown high reliability (α = 0.938) (Arias-Gundín & García, 2007). In the present study, this task also demonstrated high reliability (α = 0.890). In this task, students were presented with a text entitled The Fox and the Wolf, structured as a typical compare-and-contrast composition consisting of three paragraphs. The text includes both mechanical errors (spelling, grammar, and punctuation) and substantive errors (content repetition, disorganized elements, lack of information) in order to facilitate students’ revision. They were asked to read it, review it, and rewrite it with improvements.

Several revision measures were extracted following the categorization proposed by Chanquoy (1997). The number of mechanical-level changes that each student made to their own text, with respect to the original text provided, yielded three variables: spelling, grammar, and punctuation. At the substantive level, four variables were obtained: addition, deletion, change, and reorganization. Table 2 presents a description of each variable, accompanied by an example of a revision produced by a student. For each example, the original text is shown first, followed by the version modified by the student.

### 2.4. Instructional Programs

For the present study, three versions of the instructional program were designed: (a) one focused exclusively on mechanical aspects, (b) another targeting substantive aspects, and (c) a combined modality integrating both. Given that most previous studies have focused primarily on mechanical components (e.g., Beal, 1996; Fitzgerald & Markham, 1987; MacArthur et al., 1991), it is not only necessary to replicate these findings in Spanish, but also to conduct a more in-depth comparative analysis of both mechanical and substantive components.

To address the individual needs and characteristics of each student, the program was grounded in the framework of dynamic assessment and incorporated four progressively supportive levels of scaffolding, thereby promoting self-regulation (Chanquoy, 2001; Hayes et al., 1987). Level 1 involved minimal assistance, focused on guided rewriting through self-questioning. Level 2 introduced a metacognitive revision guide. Level 3 provided students with a comprehensive list of strategies to support informed selection. Fiinally, Level 4, representing maximum support, offered a rewritten version of the text with all strategies already applied, so that students could compare, adjust, and redraft.

All the texts used throughout the sessions were drawn from original productions written by students of the same age who had participated in a writing workshop during the previous year, in line with earlier studies (Chanquoy, 2001). All texts were comparable length and contained a similar number and type of errors; where necessary, mistakes were deliberately introduced, following the methodology employed in previous research (Cameron et al., 1997).

The overall instructional sequence for each session comprised the following components: (1) the activation and consolidation of prior knowledge, based on examples provided by the students themselves; (2) joint instruction, delivered in an interactive style that directed attention towards key content; and (3) the rewriting of a text using the CDO procedure (careful reading, error detection through underlining, generation of improvement proposals, and rewriting), with each student employing the revision guide and rewriting sheet corresponding to the level of support they required. Taken together, these elements aimed to foster strategic and self-regulated learning in relation to students’ metacognitive understanding of the textual revision process.

Below, we provide a concise description of each session included in the program.

Session 1 begins by fostering students’ motivation regarding the relevance of effective writing. To this end, a brainstorming activity is conducted in which students identify concrete, real-life situations in which they consider writing to be important, thereby encouraging them to recognize new perspectives. The session continues with a review of the cognitive processes involved in writing and planning. This leads to a guided discussion on the processes students typically activate when composing a text, after which the PER strategy [from its Spanish initials: **P**lanificar (planning), **E**ditar (transcription), and **R**evisar (revising)] is introduced to support recall and promote strategic awareness. With respect to the planning process, the instructor provides a brief explanation of its component subprocesses through a think-aloud modelling exercise, using an example aligned with students’ interests. These subprocesses are presented through the RODIO strategy: **R**ecoger información (gather information: activating prior knowledge on the topic), **O**bjetivo (aim: clarifying the purpose of the text), **D**estinatario (audience: identifying the intended audience), **I**deas principales (main ideas: determining the key ideas to be developed), and **O**rganización (organization: structuring and sequencing these ideas). To conclude the session, students are asked to revise and rewrite an assigned text. They are instructed to begin by reading the text carefully, identifying potential errors, and subsequently producing an improved version on a rewriting sheet that contains a set of guiding self-questions. Although the content of this sheet varies according to the specific modality of the program, all versions include a shared initial self-instruction: “I make an effort to pause and check how my text is turning out.” Table 3 provides the specific content related to the mechanical and substantive aspects of revision, which are addressed separately in the programs focusing on each component. In the combined program, both types of content are integrated and taught jointly.

In the second session, students will begin an intuitive exploration of the textual genre they will work with throughout the program: comparison–contrast text. Through a guided discussion, they will analyze an exemplary model entitled Animated and Adventure Films. Following this, the instructor will summarize the defining features of a comparison–contrast text, highlighting its main characteristics and specifying the content that should be included in each of the six constituent paragraphs, as detailed in Table 4. To conclude the session, students will be tasked with revising and rewriting a new text. Prior to commencing this activity, they will be introduced to the CDO procedure (Scardamalia & Bereiter, 1983, 1985). In this session, the self-questions embedded in the rewriting worksheet will be organized by paragraph. Consistent with the first session, their content will focus specifically on the mechanical or substantive aspects of revision, depending on the program modality, or on both in the case of the combined program.

Session 3 focuses on developing students’ metacognitive knowledge of the revision process. Declarative knowledge will be introduced based on the information provided by students in response to the questions “What do you revise in a text?” and “Why do you do so?”. Procedural and conditional knowledge will be addressed through the CDO procedure (Compare, Diagnose, Operate): for Comparing, students will be asked to read the text carefully and consider whether there is anything incorrect or in need of improvement; for Diagnosing, they will underline the fragment in which they have identified a problem and reflect on how it might be resolved; finally, they must Operate by proposing specific improvements. To support this process, students will be provided with a metacognitive revision guide organized into three columns: the first lists the aspects to be revised (mechanical, substantive, or both) depending on the program modality; the second requires students to identify the problem detected in the text; and the third asks them to propose how they would improve it. In this session, the guide includes only the aspects to be revised and their definitions, whereas in the following session it will be expanded to incorporate self-questions, as shown in Table 5. As in all sessions, it will conclude with the revision and rewriting of a text.

During Session 4, instructional content introduced in the previous session is further consolidated through the presentation of a detailed revision guide. This instrument is designed to support students in recalling and accurately identifying each evaluative category, facilitated by the use of structured self-question prompts. Instructional scaffolding is provided through response modelling: erroneous responses are progressively shaped until the target response is achieved, whereas accurate responses receive immediate reinforcement. Prior to initiating the text-rewriting task, students are reminded of the sequential steps embedded within the CDO procedure to ensure procedural fidelity.

Session 5 focuses on delineating the distinction between rewriting a text and simply reproducing it with isolated error corrections. Students are provided with a text entitled Music and instructed to read it attentively, identify and mark errors, and underline the principal ideas. The instructor subsequently emphasizes that text rewriting does not entail copying the original text while correcting the highlighted errors; instead, it requires generating a new version based on the previously extracted main ideas. To render this distinction explicit, students are supplied with two versions of the Music text: one fully rewritten and one merely copied with corrections.

Session 6 aims to strengthen students’ mastery of text-rewriting processes and to reinforce the components of the CDO procedure and the associated revision guide. This consolidation phase ensures greater internalization of both metacognitive monitoring strategies and procedural steps.

The final three sessions are devoted to comprehensive revision and repeated practice of all previously taught strategies, with particular emphasis on applying the CDO procedure to the rewriting of complete texts. Individualized scaffolding is provided to each student according to their assessed level of need, delivered via tailored support materials.

### 2.5. Procedure

The study was conducted within the four second-year compulsory secondary education classes of a high school in León. Initial contacts with the school were established in the previous academic year with the headteacher and deputy head, who formally authorised the intervention. At the start of the year, planning meetings were held with the deputy head and the Spanish Language teacher responsible for all four participating groups, and parental consent was obtained for student participation.

Groups were randomly assigned to the experimental conditions, regardless of the Spanish Language timetable or prior academic performance. One group was assigned to the practice condition, while the remaining three participated in different instructional program modalities.

Pretest and posttest measures were collected using the IRCE rewriting task (Arias-Gundín & García, 2007), which evaluates the number of revisions students carry out at both the mechanical and the substantive level.

The instructional program consisted of nine weekly sessions of 45–50 min, conducted on Mondays during regular class hours, with group sessions led by the first author.

At the conclusion of each session, the first author reviewed and analyzed the texts produced by the students using a dynamic-assessment-based monitoring sheet, determining the level of support required in the following session. Six performance categories were established along a continuum of complexity: (C−) Literal copying, retaining most original errors and sometimes adding new ones; (C) Copying with minimal modifications; (C+) Correction of mechanical errors without addressing substantive content; (R−) Correction of mechanical errors with some content modifications; (R) Rewriting that expands information and removes unnecessary elements; (R+) Reorganization and restructuring, including connectives, balanced paragraph development, and comparison of relevant content elements. When a student’s rewritten text received a copy rating in any of its forms, the level of support was increased in the subsequent session; when it received an R− or R rating, the level of support was maintained; and when it was rated as R+, the level of support was reduced.

After the intervention, all assessment tasks were corrected, digitized, and subjected to statistical analysis in accordance with the study’s aims.

### 2.6. Data Analysis

First, the distribution of each variable was examined, confirming that kurtosis and skewness values fell within the ranges of ±7 and ±3, respectively (Kline, 2011). The suitability of the data for parametric analyses was then assessed using the Shapiro–Wilk and Levene tests, verifying the null hypotheses that the sample data are normally distributed and that the population variances across groups are equal (homoscedasticity), respectively, with *p* > 0.05. Pearson correlation coefficients were subsequently calculated to ensure the absence of multicollinearity among the variables under study, with r > 0.50 considered indicative of a strong or very strong relationship. To verify that no statistically significant differences existed between groups at the start of the study, analyses of variance were performed on both mechanical and substantive revision variables (*p* < 0.05). Finally, to evaluate the effectiveness of the instructional programs, multivariate analyses of variance were conducted. Initially, a 4 × 2 repeated-measures factorial design was employed, with assessment moment (pre-test vs. post-test) as the within-subjects factor and treatment type (practice, mechanical revision, substantive revision, and combined revision) as the between-subjects factor. The Bonferroni test was subsequently applied to identify group differences in the variables that required post hoc analysis.

## 3. Results

As previously indicated, in order to assess the effectiveness of the intervention, the rewriting task from the Instrumento de Revisión de la Composición Escrita (IRCE, by its initials in Spanish) was employed, which enables the evaluation of the different components involved in textual revision.

First, the results concerning the mechanical aspects of revision are presented, followed by those related to substantive revision.

### 3.1. Mechanical Revision

The following section presents the results related to the variables included in mechanical revision: spelling, grammar, and punctuation.

Firstly, the suitability of the results for parametric testing was examined, in both the pre-test and the post-test, using the Shapiro–Wilk and Levene’s tests, as shown in Table 6.

Subsequently, multicollinearity among the variables was examined in both the pre-test and the posttest. As shown in Table 7, none of the correlations exceed 0.49; therefore, no variable exhibits a strong correlation.

In the preliminary analysis, no statistically significant differences were found between groups at the beginning of the program in the rewriting task at the mechanical level [λ (3, 77) = 0.898; *F* (3, 77) = 0.895; *p* = 0.531; η*p*^2^ = 0.035]. Conversely, statistically significant differences were found after the implementation of the instructional program, [λ (3, 77) = 0.751; *F* (3, 77) = 2.462; *p* = 0.011; η*p*^2^ = 0.091]. Table 8 presents the descriptive statistics by group for all variables analyzed in the study according to the assessment time.

No statistically significant differences were found for spelling [*F* (3, 77) = 2.251; *p* = 0.089; η*p*^2^ = 0.083] or grammar [*F* (3, 77) = 0.920; *p* = 0.435; η*p*^2^ = 0.035]; however, such differences were confirmed for punctuation [*F* (3, 77) = 3.067; *p* = 0.033; η*p*^2^ = 0.109]. Specifically, the post hoc contrasts showed that there were significant differences between the rewriting group and both the substantive (*p* = 0.028) and the combined (*p* = 0.022) groups.

**Figure 1:**
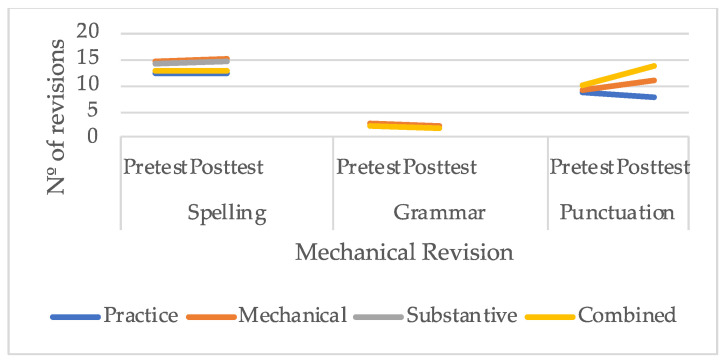
Number of mechanical revisions performed by students.

As shown in Figure 1, the number of revisions made by students in spelling and grammar hardly changed after the completion of the study. From the outset, spelling was the mechanical aspect of revision that received the greatest attention from students, as it was the area in which they produced the highest number of revisions; in contrast, grammar was the aspect that received the fewest revisions. Regarding punctuation, it can be observed that students who received instruction in substantive revision or in the combined approach were those who showed the greatest increase in the number of revisions by the end of the study.

### 3.2. Substantive Revision

The following section reports the results pertaining to the variables included in substantive revision: add, delete, change and reorganize.

Initially, the appropriateness of the data for parametric analysis was assessed for both the pretest and the posttest through the Shapiro–Wilk and Levene’s tests, as presented in Table 9.

Next, the presence of multicollinearity among the variables was evaluated for both the pretest and posttest. As indicated in Table 10, all correlations were below 0.49, indicating that no variable demonstrated a strong relationship.

In the initial analysis, no statistically significant differences were observed between groups at the start of the program in the substantive revision [λ (3, 77) = 0.776; *F* (3, 77) = 1.598; *p* = 0.095; η*p*^2^ = 0.81]. In contrast, following the implementation of the instructional program, statistically significant differences were observed in the substantive aspects of revision [λ (3, 77) = 0.688; *F* (3, 77) = 2.411; *p* = 0.006; η*p*^2^ = 0.117]. Table 11 displays the group-wise descriptive statistics for all substantive revision variables examined in the study across the different assessment points.

No statistically significant differences were found for deletion [*F* (3, 77) = 0.451; *p* = 0.718; η*p*^2^ = 0.018], change [*F* (3, 77) = 0.594; *p* = 0.621; η*p*^2^ = 0.023] and reorganize [*F* (3, 77) = 0.995; *p* = 0.400; η*p*^2^ = 0.038]. In contrast, statistically significant differences were found between groups in interaction with the instructional program for addition [*F* (3, 77) = 3.413; *p* = 0.022; η*p*^2^ = 0.120]. Specifically, the post hoc contrasts showed that there were significant differences between the rewriting group and both the substantive (*p* = 0.012) and the combined (*p* = 0.010) groups.

**Figure 2:**
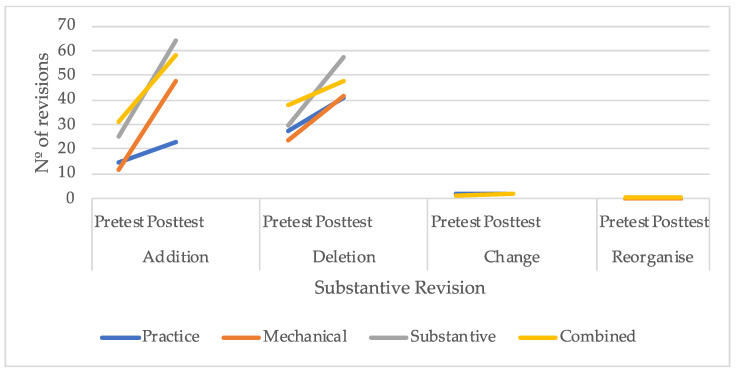
Number of substantive revisions performed by students.

Moreover, as illustrated in Figure 2, students performed very few revisions involving changes or reorganization of the text, with no discernible differences in patterns among the four groups. In contrast, statistically significant differences were observed in content addition, specifically between students in the text-rewriting practice group and those who received explicit instruction in either substantive or combined revision. Although no statistically significant differences were detected for the group receiving instruction in mechanical revision, this group demonstrated a similar trend of increased revisions to that observed in the other two instructed groups. Finally, with respect to content deletion, while no statistically significant differences emerged between groups, participants in any of the instructional programs exhibited a pattern of greater increases compared to the text-rewriting practice group.

## 4. Discussion

The findings of this study provide empirical support for the effectiveness of self-regulated instruction in enhancing the textual revision process among secondary school students. Consistent with prior research, the results highlight that novice writers who do not receive explicit instruction tend to engage in few revisions and focus almost exclusively on superficial corrections (García & Arias-Gundín, 2004; Scott & Vitale, 2003). Within this framework, the present study offers an initial contribution: differentiated, explicit, and scaffolded instruction constitutes an effective pedagogical resource for increasing the number of revisions undertaken by students.

Concerning the first hypothesis, which anticipated that a program focused on mechanical aspects would generate a greater number of revisions in spelling, grammar, and punctuation, given that students already possess declarative knowledge in these areas but may lack the procedural or conditional knowledge required to activate the revision process, the results do not support this expectation. No significant differences or clear improvement trends were observed in spelling or grammar revisions between groups. This pattern may be explained by a possible ceiling effect, as these skills are typically well consolidated by secondary education. Regarding punctuation, statistically significant differences were identified between the group that practiced text rewriting and those that received substantive or combined instruction. Although the mechanical revision group did not differ significantly from the rewriting group, it did show an increase in the number of revisions. Overall, the data suggest that any form of instruction directed at the revision process facilitates attention to punctuation. Moreover, as the greatest improvements were observed in groups receiving substantive instruction, it may be argued that punctuation is closely linked to overall coherence and textual structure rather than purely mechanical aspects. However, these interpretations should be approached with caution given the exploratory nature of the study. Future research should include tasks specifically designed to evaluate the detection and correction of mechanical errors, as well as their impact on longer texts, to further examine the relationship between punctuation, coherence, and textual quality.

The second hypothesis, proposing that instruction focused on substantive aspects would facilitate the activation of the revision process and increase modifications aimed at reorganizing, adding, deleting, or changing content, could also not be fully confirmed. No significant differences were observed in revisions related to reorganization or content modification across groups. However, significant differences were found in the addition category, particularly between the rewriting group and those receiving instruction on substantive aspects of revision. Although these differences were not statistically significant in the group working solely on mechanical aspects, a similar trend was evident. Similarly, no significant differences emerged in the deletion category, yet a consistent trend was observed: all instructional groups increased the number of deletions. These findings may indicate an evolutionary pattern in the development of substantive revision skills during adolescence. Students appear to first develop the ability to add content, followed by the capacity to remove redundant or irrelevant information, while revisions involving reorganization or content modification, which are cognitively more demanding, remain less frequent. Given the methodological limitations of the study, these results should be interpreted cautiously. Future research should assess these skills in isolation and subsequently in longer texts, both researcher-designed and student-produced, to explore potential divergences in strategy implementation depending on text type.

The third hypothesis, positing that the combined program would be the most effective by integrating instruction in both mechanical and substantive aspects, was also not confirmed. Data indicate that the two programs targeting substantive aspects were equally effective and outperformed the other modalities. These results reinforce the notion that instruction focused on substantive components promotes a more global, balanced, and iterative approach to revision, consistent with contemporary models of the writing process (Graham, 2018; Hayes, 2012). Furthermore, they demonstrate that this type of instruction facilitates the activation of metacognitive and self-regulatory processes necessary for deep revision, beyond superficial or linear changes.

Another contribution of the study lies in the incorporation of dynamic assessment principles, which allow scaffolding to be adjusted according to students’ individual needs. The results suggest that this approach serves a formative function by enhancing self-regulation and strategic decision-making during revision (Lidz & Elliott, 2000; Poehner, 2008). This is particularly relevant for students with fewer opportunities to develop revision strategies in traditional educational contexts.

As previously noted, this study is exploratory and presents certain limitations. Firstly, the sample was drawn from a single educational center, with only one class per condition, limiting the generalizability of the results, although instruction in the Spanish Language area was consistently delivered by the same teacher. Secondly, only short-term effects were evaluated, precluding assessment of the sustainability of learning; moreover, the transfer of results to other textual genres was not examined. Additionally, the study focused on the number of revisions made by students, without evaluating the impact of these revisions on text quality. Finally, while the IRCE task provided a reliable measure, a blind double-scoring system was not employed, and evaluation was restricted to the product; this underscores the need for future studies to incorporate process-oriented measures, such as smartpens or think-aloud protocols, to gain a deeper understanding of students’ real-time revision decision-making.

Despite these limitations, the present study contributes to the field by demonstrating that strategic instruction, accompanied by tailored and differentiated support, can significantly enhance students’ revision skills, strengthening both their writing competence and their capacity for self-regulation.

## 5. Conclusions

The present study provides robust evidence on the effectiveness of a self-regulated instructional program in text revision, designed within the framework of dynamic assessment and with a differentiated emphasis on the mechanical and substantive levels of revision. Although improvements were observed only in punctuation at the mechanical level, likely because secondary school students already possess well-consolidated declarative knowledge, substantive revisions increased markedly, particularly in the groups receiving targeted instruction in substantive revision or working under the combined modality. These findings highlight the importance of explicit and structured guidance in promoting deeper, more reflective, and strategic revision processes, going beyond the superficial corrections typically observed in novice writers.

A key theoretical contribution of this research is the reinforcement of the conception of revision as a complex, self-regulated process that benefits from progressive instruction tailored to students’ needs. Methodologically, the integration of dynamic assessment proved instrumental in adjusting the level of support for each student, promoting gradual progression towards autonomy and strategic engagement in the revision process. The differentiation in revision patterns, where substantive instruction led to more evenly distributed attention across the text, emphasizes the relevance of guiding students not only on what to revise but also on where and how to intervene.

From an educational perspective, the study demonstrates the potential of integrating self-regulated and dynamically assessed revision programs into the secondary school curriculum. Such programs can enhance both textual competence and transferable self-regulated learning skills, equipping students with strategies applicable across diverse academic and professional contexts. Future research should extend this work to include different age groups, text genres, and teaching settings, as well as investigate the long-term retention and transfer of revision strategies.

In conclusion, strategic, self-regulated instruction, supported through adaptive scaffolding, constitutes an effective approach for improving students’ revision practices, fostering reflective writing, and developing metacognitive and self-regulatory skills essential for lifelong learning.

## Figures and Tables

**Table 1:** Demographic distribution of the study sample by age, group, and gender.

	Group	Practice Rewriting	Experimental Mechanical Revision	Experimental Substantive Revision	Experimental Combined Revisions	Total Gender
**Gender**	Male	11 (9)	13 (11)	9 (7)	10 (10)	43 (37)
	Female	10 (9)	10 (9)	14 (13)	11 (11)	45 (42)
**Total group**		21 (18)	23 (20)	23 (20)	21 (21)	88 (79)
**Average age by group**		13.66 (13.67)	13.30 (13.35)	13.43 (13.40)	13.28 (13.28)	
**min-max age**		13–15 (13–15)	13–14 (13–14)	13–15 (13–15)	13–15 (13–15)	

Note: In parentheses, the final sample considered for conducting the statistical analyses is indicated.

**Table 2:** Description of Revision Measures with Student Examples.

Variables	Description	Original Text	Student Revision Example
**Mechanical Revision**			
**Spelling**	Word spelled incorrectly according to official orthographic rules	El lobo es un **mamifero**… [*The wolf is a mammal…*] . **su** estatura es grande, *[.its size is large,]*	El lobo es un mam**í**fero… [*The wolf is a mammal…*] . **S**u estatura es grande, *[. Its size is large,*]
**Grammar**	Incorrect use of structures, verb tenses, agreement, or pronouns.	El lobo y el zorro se parecen… **son carnivoro**… [*The wolf and the fox are similar… they are carnivore…*]	El lobo y el zorro se parecen… son carnivoro**s**… [*The wolf and the fox are similar… they are carnivores…*]
**Punctuation**	Incorrect use of punctuation marks	… el lobo es **carnívoro es pequeño y de pelaje oscuro el ocico** es puntuagudo… *[…the wolf is carnivorous is small and has dark fur its snout is pointed…*]	… el lobo es carnívoro, pequeño y de pelaje oscuro. El ocico es puntuagudo… [*…the wolf is carnivorous, small, and has dark fur. Its snout is pointed…*]
**Substantive Revision**			
**Addition**	Add new content, which may consist of a single word	El zorro es un mamífero y come carne *[The fox is a mammal and eats meat]*	El zorro es un mamífero y come carne, **por ejemple ovejas** *[The fox is a mammal and eats meat, for example sheep]*
**Deletion**	Delete existing content, which may consist of a single word	… el lobo **es carnívoro es pequeño** y de pelaje oscuro… *[…the wolf is carnivorous is small and has dark fur…*]	… el lobo es carnívoro, pequeño y de pelaje oscuro… [*…the wolf is carnivorous, small, and has dark fur…*]
**Change**	Replace one item with another that represents the same meaning	…, y hasta cuando tiene mucha hambre **ataca al hombre**. […and when it is very hungry, it may even attack person.]	…, y hasta cuando tiene mucha hambre **come humanos**. […and when it is very hungry, it may even eat humans.]
**Reorganized**	Reorder the content of the text, either at the sentence level or at the paragraph level	El zorro es un mamífero y **come carne** igual que el lobo… y **come aves de caza**. *[The fox is a mammal and eats meat like the wolf… and eats game birds.]*	El zorro es un mamífero y **come carne por ejemplo aves de caza…** *[The fox is a mammal and eats, as well as game birds.]*

Note: Words or set of words in which an error has been identified are indicated in bold.

**Table 3:** Content of the self-instructions according to the revision program modality.

Mechanical Revision	Substantive Revision
Before introducing a new idea, it is important to pause and carefully read what has been written:	
Is my handwriting clear and legible? Are the paragraphs appropriately separated? Are all words spelled correctly? Is punctuation used correctly throughout the text? Are verb tenses accurate? Do nouns and adjectives agree in gender and number where applicable?	Does the text accurately convey my intended meaning? Is the content clear and engaging? Are ideas logically organised, or are they mixed together? Have I included all relevant information for this idea? Can additional content be added, or should any material be removed? Are there words or expressions that are unnecessarily repeated?

**Table 4:** Definition and Structure of a Comparison–Contrast Text.

**Definition of the Textual Genre**	This structure corresponds to texts in which the author’s aim is to compare two topics, specifying their similarities in the case of comparison and their differences in the case of contrast.
**Structure**	
Paragraph 1	Introduction presenting the topic and purpose.
Paragraph 2	Description of the first element being compared.
Paragraph 3	Description of the second element being compared.
Paragraph 4	Discussion of the similarities between the two elements.
Paragraph 5	Discussion of the differences between the two elements.
Paragraph 6	Conclusions and personal opinion.

**Table 5:** Framework of Revision Components: Definitions and Self-Regulatory Self-Questions.

Mechanical Aspects		Substantive Aspects	
Session 3	Session 4	Session 3	Session 4
**External appearance:** formal presentation of the text, cleanliness, neat handwriting, and overall layout.	Is my handwriting clear and legible? Have I separated paragraphs appropriately? Have I respected the margins? Is my text presented neatly?	**Word substitution:** use of synonyms to replace repeated words and/or expressions.	Are there repeated words? What synonyms can I use to replace those words and/or expressions?
**Spelling:** correct application of spelling conventions.	Have I used capital letters correctly? Have I spelled all words correctly? Have I included all required accent marks?	**Addition of content:** expanding the text by incorporating relevant aspects, ideas, or details.	Have I forgotten any idea? Are additional details necessary?
**Punctuation:** correct use of punctuation rules.	Have I punctuated my text clearly so that it is easy to understand?	**Deletion of material:** removing aspects, ideas, or details that are irrelevant or repeated.	Should I delete any word or expression? Is there anything unnecessary in my text?
**Grammar:** correct use of grammatical rules, including function words and agreement.	Do all subjects agree with their verbs? Have I used verb tenses correctly? Do nouns agree with their articles and adjectives in gender and number?	**Reordering:** reorganizing and restructuring the content of the text.	Is the way I begin the text appropriate? Am I mixing ideas?

**Table 6:** Suitability of the mechanical revision data for parametric testing using the Shapiro–Wilk and Levene’s tests.

Variables	Group	Pretest		Posttest	
		Shapiro–Wilk Test	Levene Test	Shapiro–Wilk Test	Levene Test
**Spelling**	Practice	*W*(18) = 0.954, *p* = 0.491		*W*(18) = 0.939, *p* = 0.280	
	Mechanical	*W*(20) = 0.926, *p* = 0.130		*W*(20) = 0.946, *p* = 0.313	
	Substantive	*W*(20) = 0.968, *p* = 0.708		*W*(20) = 0.909, *p* = 0.060	
	Combined	*W*(21) = 0.932, *p* = 0.152		*W*(21) = 0.970, *p* = 0.731	
	Total		*F*(3,75) = 1.075, *p* = 0.372		*F*(3,75) = 1.381, *p* = 0.183
**Grammar**	Practice	*W*(18) = 0.976, *p* = 0.881		*W*(18) = 0.952, *p* = 0.152	
	Mechanical	*W*(20) = 0.967, *p* = 0.707		*W*(20) = 0.940, *p* = 0.237	
	Substantive	*W*(20) = 0.958, *p* = 0.498		*W*(20) = 0.925, *p* = 0.125	
	Combined	*W*(21) = 0.912, *p* = 0.070		*W*(21) = 0.908, *p* = 0.090	
	Total		*F*(3,75) = 0.675, *p* = 0.570		*F*(3,75) = 2.217, *p* = 0.093
**Punctuation**	Practice	*W*(18) = 0.955, *p* = 0.517		*W*(18) = 0.971, *p* = 0.819	
	Mechanical	*W*(20) = 0.955, *p* = 0.453		*W*(20) = 0.913, *p* = 0.087	
	Substantive	*W*(20) = 0.970, *p* = 0.750		*W*(20) = 0.903, *p* = 0.079	
	Combined	*W*(21) = 0.919, *p* = 0.084		*W*(21) = 0.920, *p* = 0.096	
	Total		*F*(3,75) = 1.193, *p* = 0.318		*F*(3,75) = 0.133, *p* = 0.940
**Total Mechanical**	Practice	*W*(18) = 0.953, *p* = 0.468		*W*(18) = 0.937, *p* = 0.253	
	Mechanical	*W*(20) = 0.903, *p* = 0.063		*W*(20) = 0.959, *p* = 0.522	
	Substantive	*W*(20) = 0.962, *p* = 0.579		*W*(20) = 0.919, *p* = 0.095	
	Combined	*W*(21) = 0.957, *p* = 0.458		*W*(21) = 0.920, *p* = 0.086	
	Total		*F*(3,75) = 2.164, *p* = 0.099		*F*(3,75) = 2.617, *p* = 0.082

**Table 7:** Correlations of the mechanical revision variables according to assessment time.

Variables	Pretest		Posttest	
	1.	2.	1.	2.
**1. Spelling**				
**2. Grammar**	0.475 **		0.200	
**3. Puctuation**	0.438 **	0.065	0.432 **	−0.127

Note: ** *p* < 0.01.

**Table 8:** Descriptive statistics by group for the mechanical revision variables in the study according to assessment time.

Variables	Practice Rewriting		Mechanical Revision		Substantive Revision		Combined Revision	
	M Pre (SD)	M Post (SD)	M Pre (SD)	M Post (SD)	M Pre (SD)	M Post (SD)	M Pre (SD)	M Post (SD)
**Spelling**	12.44 (5.84)	12.22 (6.45)	14.65 (1.21)	14.95 (3.35)	13.90 (4.61)	14.35 (3.83)	12.76 (3.69)	12.62 (3.77)
**Grammar**	2.17 (0.92)	2.33 (1.28)	2.70 (0.86)	2.20 (1.39)	2.30 (0.92)	1.90 (1.16)	2.19 (0.81)	1.81 (0.81)
**Punctuation**	8.61 (4.68)	7.72 (4.13)	8.95 (3.00)	10.90 (4.25)	9.90 (4.27)	13.65 (5.76)	10.24 (5.27)	13.43 (4.72)
**Total Mechanical**	23.22 (10.30)	21.67 (20.41)	26.30 (5.93)	24.65 (6.89)	26.10 (7.67)	29.50 (8.64)	25.19 (7.77)	27.62 (6.09)

**Table 9:** Suitability of the substantive revision data for parametric testing using the Shapiro–Wilk and Levene’s tests.

Variables	Group	Pretest		Posttest	
		Shapiro–Wilk Test	Levene Test	Shapiro–Wilk Test	Levene Test
**Addition**	Practice	*W*(18) = 0.928, *p* = 0.132		*W*(18) = 0.903, *p* = 0.072	
	Mechanical	*W*(20) = 0.955, *p* = 0.452		*W*(20) = 0.941, *p* = 0.283	
	Substantive	*W*(20) = 0.945, *p* = 0.293		*W*(20) = 0.910, *p* = 0.074	
	Combined	*W*(21) = 0.929, *p* = 0.137		*W*(21) = 0.954, *p* = 0.397	
	Total		*F*(3,75) = 1.111, *p* = 0.324		*F*(3,75) = 2.669, *p* = 0.064
**Deletion**	Practice	*W*(18) = 0.972, *p* = 0.837		*W*(18) = 0.952, *p* = 0.463	
	Mechanical	*W*(20) = 0.975, *p* = 0.879		*W*(20) = 0.917, *p* = 0.088	
	Substantive	*W*(20) = 0.976, *p* = 0.881		*W*(20) = 0.911, *p* = 0.068	
	Combined	*W*(21) = 0.964, *p* = 0.603		*W*(21) = 0.977, *p* = 0.883	
	Total		*F*(3,75) = 0.299, *p* = 0.826		*F*(3,75) = 1.461, *p* = 0.232
**Change**	Practice	*W*(18) = 0.929, *p* = 0.134		*W*(18) = 0.915, *p* = 0.132	
	Mechanical	*W*(20) = 0.957, *p* = 0.461		*W*(20) = 0.968, *p* = 0.293	
	Substantive	*W*(20) = 0.971, *p* = 0.808		*W*(20) = 0.902, *p* = 0.078	
	Combined	*W*(21) = 0.946, *p* = 0.204		*W*(21) = 0.948, *p* = 0.169	
	Total		*F*(3,75) = 1.096, *p* = 0.356		*F*(3,75) = 0.720, *p* = 0.543
**Reorganize**	Practice	*W*(18) = 0.952, *p* = 0.502		*W*(18) = 0.950, *p* = 0.293	
	Mechanical	*W*(20) = 0.936, *p* = 0.372		*W*(20) = 0.946, *p* = 0.258	
	Substantive	*W*(20) = 0.957, *p* = 0.456		*W*(20) = 0.923, *p* = 0.208	
	Combined	*W*(21) = 0.901, *p* = 0.152		*W*(21) = 0.908, *p* = 0.162	
	Total		*F*(3,75) = 0.930, *p* = 0.430		*F*(3,75) = 1.437, *p* = 0.239
**Total Substantive**	Practice	*W*(18) = 0.926, *p* = 0.166		*W*(18) = 0.907, *p* = 0.076	
	Mechanical	*W*(20) = 0.936, *p* = 0.084		*W*(20) = 0.912, *p* = 0.083	
	Substantive	*W*(20) = 0.979, *p* = 0.926		*W*(20) = 0.904, *p* = 0.059	
	Combined	*W*(21) = 0.916, *p* = 0.072		*W*(21) = 0.963, *p* = 0.575	
	Total		*F*(3,75) = 1.075, *p* = 0.365		*F*(3,75) = 0.583, *p* = 0.628

**Table 10:** Correlations of the substantive revision variables according to assessment time.

Variables	Pretest			Posttest		
	1.	2.	3.	1.	2.	3.
**1. Addition**						
**2. Deletion**	0.450 **			0.499 **		
**3. Change**	−0.142	−0.168		0.399 **	0.412 **	
**4. Reorganize**	0.099	0.187	0.207	0.083	0.189	0.182

Note: ** *p* < 0.01.

**Table 11:** Descriptive statistics by group for the substantive revision variables in the study according to assessment time.

Variables	Practice Rewriting		Mechanical Revision		Substantive Revision		Combined Revision	
	M Pre (SD)	M Post (SD)	M Pre (SD)	M Post (SD)	M Pre (SD)	M Post (SD)	M Pre (SD)	M Post (SD)
**Additon**	14.50 (14.79)	22.67 (20.31)	11.65 (12.76)	47.90 (43.75)	25.15 (14.16)	64.10 (45.36)	31.00 (26.32)	58.43 (36.12)
**Deletion**	27.06 (21.96)	40.78 (26.25)	23.35 (19.82)	41.90 (27.77)	30.00 (23.62)	57.20 (18.99)	37.62 (18.52)	47.86 (24.34)
**Change**	1.67 (1.91)	1.94 (1.47)	1.25 (1.21)	1.90 (1.45)	1.10 (1.45)	2.20 (1.96)	1.14 (1.39)	1.62 (1.53)
**Reorganize**	0.22 (0.73)	0.37 (0.91)	0.10 (0.45)	0.05 (0.22)	0.45 (0.83)	0.45 (0.76)	0.29 (0.64)	0.24 (0.54)
**Total Substantive**	43.33 (32.30)	66.06 (44.63)	46.35 (30.56)	91.75 (67.38)	56.70 (34.51)	123.95 (55.54)	60.05 (40.99)	108.14 (55.54)

## Data Availability

The raw data supporting the conclusions of this article will be made available by the authors upon request to the corresponding author, without restrictions.

## Abbreviation

The following abbreviation is used in this manuscript:

IRCE	Spanish acronym of Instrumento de Revisión de la Composición Escrita (Written Composition Revision Instrument)
